# Ultrasound-Guided Dry Needling for Trigger Point Inactivation in the Treatment of Postherpetic Neuralgia Mixed with Myofascial Pain Syndrome: A Prospective and Controlled Clinical Study

**DOI:** 10.1155/2022/2984942

**Published:** 2022-08-02

**Authors:** Yifa Huang, Mintai Gao, Qiaomin Li, Xuzheng Zhang, Huizhen Chen, Xinglu Li, Ping Hu, Qingshi Zeng

**Affiliations:** ^1^Department of Anesthesiology, Guangdong Provincial People's Hospital Zhuhai Hospital (Zhuhai Golden Bay Center Hospital), Zhuhai, Guangdong 519040, China; ^2^Department of Operation Anesthesiology, The Fifth Affiliated Hospital of Sun Yat-sen University, Zhuhai, Guangdong 519000, China; ^3^Department of Anesthesia Surgery Department, Guangdong Provincial People's Hospital Zhuhai Hospital (Zhuhai Golden Bay Center Hospital), Zhuhai, Guangdong 519040, China; ^4^Department of Anesthesiology, Guangdong Cardiovascular Institute, Guangzhou, China; ^5^Guangdong Provincial People's Hospital, Guangdong Academy of Medical Sciences, Guangzhou, Guangdong 510030, China

## Abstract

**Objective:**

To evaluate the safety and effectiveness of ultrasound-guided dry needling for trigger point inactivation in the treatment of postherpetic neuralgia (PHN) mixed with myofascial pain syndrome (MPS).

**Methods:**

A prospective and controlled clinical study was conducted. From January 2020 to December 2020, among the 100 patients who received PHN treatment in the pain department, 54 patients complicated with MPS were randomly divided into the dry needling group D (*n* = 28) and pharmacotherapeutic group P (*n* = 26). Visual analogue score (VAS) and McGill Pain Questionnaire (MPQ) were taken as primary indicators. Ultrasound-guided inactivation of myofascial trigger points (MTrPs) with dry needling and intradermal needling combined with press needling were applied on group D and pharmacotherapeutic only treatment on group P respectively. The VAS score <3 and/or the MPQ score <2 represents effective treatment. The VAS score >3 and/or the MPQ score >2 represents recurrent in follow-up study three months after the treatment.

**Results:**

After four weeks treatment, the effective rate of one month later of the group D was 92.9% and the effective rate of group P was 38.5%, respectively. The recurrent rate of group D was 7.1% and 34.6% for group P, respectively, for follow-up three months later. The satisfactory rate of group D was higher than that of group P.

**Conclusion:**

Ultrasound-guided dry needling and intradermal needling combined with press needling were more effective than only pharmacotherapeutic treatment for PHN mixed with MPS, with lower recurrent rate and higher patient's satisfactory rate.

## 1. Introduction

The annual incidence rate of herpes zoster is 0.3–0.5%, among which 9–34% of patients have postherpetic neuralgia (PHN) [1, 2]. Many studies reported that compared with other types of pain, PHN as a kind of neuropathic pain, the nerve changes can lead to persistent and severe breakthrough pain and serious harm to the patients quality of life [3, 4]. The treatment mainly relies on anticonvulsants, antidepressants, topical therapies, including oral NSAIDs, and local lidocaine and capsaicin, even injection of opioids, but the effect is not very satisfactory [1, 5, 6]. Today, the routine method for treating PHN is oral drugs and paravertebral nerve block at corresponding spinal cord segments with herpes distribution. But multiple clinical manifestations lead to irregular effects, especially in patients with MPS. Dry needling (DN) treatment is a safe technique, which leaves no side effects other than a slight discomfort that disappears after 3 days at the most [7]. Some research found out that more than a half of PHN patients had mild to moderate pain mixed with MPS [8]. MPS is also found in other conditions, such as osteoarthritis of the knee [9]. These PHN patients not only have skin trigger pain in the initial herpes distribution area but also obvious tenderness points in the distance. These patients are diagnosed with PHN mixed with MPS. MPS is a common clinical pain syndrome, and its major characteristic is local tenderness accompanied by obvious muscle tension, in which pressing can lead to autonomic nerve phenomenon. Unfortunately, there is no significant treatment [10]. Therefore, the purpose of this study is to observe the safety and effectiveness of ultrasound-guided DN in the treatment of PHN mixed with MPS.

## 2. Data and Methods

### 2.1. Study Design

The experimental methods of this study were designed in accordance with the Declaration of Helsinki. The clinical trial was registered under no. ChiCTR2100052586 in the Chinese Clinical Trial Registry, and written informed consent for pain treatment was obtained before treatment, and this study was approved by the Ethics Committee of Guangdong Provincial People's Hospital Zhuhai Hospital (no. GDREC2019018(R2)H).

### 2.2. Data

#### 2.2.1. Inclusion Criteria

**Figure 1:**
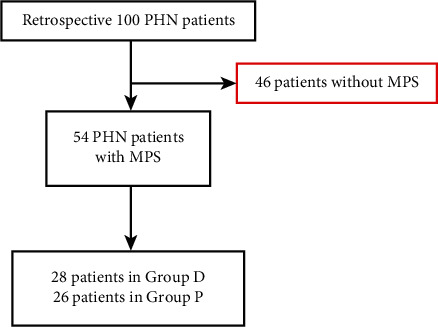
Flowchart of study inclusion and exclusion.

From January 2020 to December 2020, a total of 100 PHN patients met the preliminary screening criteria, including 46 patients without MPS and 54 patients with MPS selected for the study in the Pain Department, Guangdong Provincial People's Hospital Zhuhai Hospital, Zhuhai Golden Bay Center Hospital, and the Fifth Affiliated Hospital of Sun Yat-sen University. Before being enrolled in the study, all patients underwent necessary corresponding laboratory tests and ultrasound and CT imaging examination to exclude pain caused by medical, surgical, and gynecological diseases. These patients aged 42–88 and were clearheaded with no communication disorder. The flowchart of study inclusion and exclusion is shown in Figure 1.

#### 2.2.2. Exclusion Criteria

The exclusion criteria are as follows: obvious abnormal coagulation function; mental disorder and/or disturbance of consciousness; digestive tract obstruction or inability to take oral medicine; serious cardio or cerebrovascular disease and/or heart, liver, kidney, and lung failure, allergic to drugs used in this trial; and fainting during needling and/or unable to accept other treatments in this study.

#### 2.2.3. Patients Data

54 patients mixed with PHN and MPS were randomly divided the dry needling group (group D, *n* = 28) and pharmacotherapeutic group (group P, *n* = 26), and the patients characteristics are given in Table 1, and there were no significance difference between the two groups (*p* > 0.05).

### 2.3. Treatment Methods and Effect Evaluation

#### 2.3.1. Treatment Methods

Group P: the patients took pregabalin (Pfizer Pharmaceutical Co., Ltd., 75 mg/tablet) twice a day and mecobalamin (Weicai Pharmaceutical Co., Ltd., 0.5 mg/tablet) three times a day for 4 weeks. Group D: ultrasound-guided DN combined with intradermal needling and united with press needling once a week for 4 weeks. The body was examined in advance by whole target assessment: the standard standing posture of human anatomy was adopted, with the sagittal line as the baseline and the scapula as the reference substance. If the scapula rotates clockwise, the preliminary target muscles were left internal oblique and latissimus dorsi and right external oblique and pectoralis major. If the scapula rotates counter clockwise, the opposite was true. Then, the corresponding locations were examined with a high-frequency probe and the MTrPs were marked after examination. The skin was sterilized by 75% alcohol and placed with a sterile surgical towel. The ultrasonic probe was covered with a sterile sleeve, and the marking point was located in the center of the ultrasonic probe. The intraoperative validation of external abdominal oblique muscle was referred to the study of Sanchez Romero et al. [11]. The MTrPs were punctured with stainless steel filiform needle on the real-time ultrasound-guided in-plane technique to induce obvious aching and distending pain, if local twitch responses (LTRs) could been induced, conducting repeated stimulation until LTRs could not be induced. The pain points of local pressing were treated with intradermal needling. The needling set was Huatuo brand stainless steel filiform needle (specification: 0.3^*∗*^50 mm), and the intradermal needling was Huaqiu brand stainless steel needling (specification: 0.3^*∗*^0.5 mm and 0.15^*∗*^0.2 mm).

#### 2.3.2. Effective Evaluation

Visual analogue scale (VAS) and McGill Pain Questionnaire (MPQ) were recorded before treatment. Characteristics such as ASA grade, age, gender, mixed diseases, height, weight, and pulse were also recorded in both groups. After each treatment, the VAS and MPQ were conducted and recorded.

1. Evaluation tool: pain assessment was performed by specialized nurses after each treatment, the critical score including VAS score and MPQ score. If the VAS score <3 and/or the MPQ score <2, effective treatment was recognized; if not, the treatment was defined as ineffective, and the effective rate was calculated. If the patient thought that the pain control did not meet the expectation, we gave analgesic drugs and recorded a remedy. Three months after the end of treatment cycles, follow-up was conducted by telephone to find out the recurrence. The VAS score >3 or the MPQ score >2 was regarded as representing recurrence.
2. VAS: the VAS was a 10 cm horizontal line labeled no pain with “Zero” and the most severe pain with “Ten.” The patients were asked to mark on this line where the intensity of the pain score. The VAS was a simple and effective method, which was usually used clinically to evaluate the degree of pain.
3. Evaluation of MTP by real-time ultrasound: the real-time ultrasound can observe the size of MTrPs' changes; if the MTrPs became smaller than the original ones, the treatment was effective; otherwise, the treatment was ineffective.
4. Adverse reactions: the adverse reactions related to treatment were completely recorded, including drowsiness, nausea, vomiting, subcutaneous bleeding, allergic reactions, panic, and dizziness.

#### 2.3.3. Statistical Analysis

**Figure 2:**
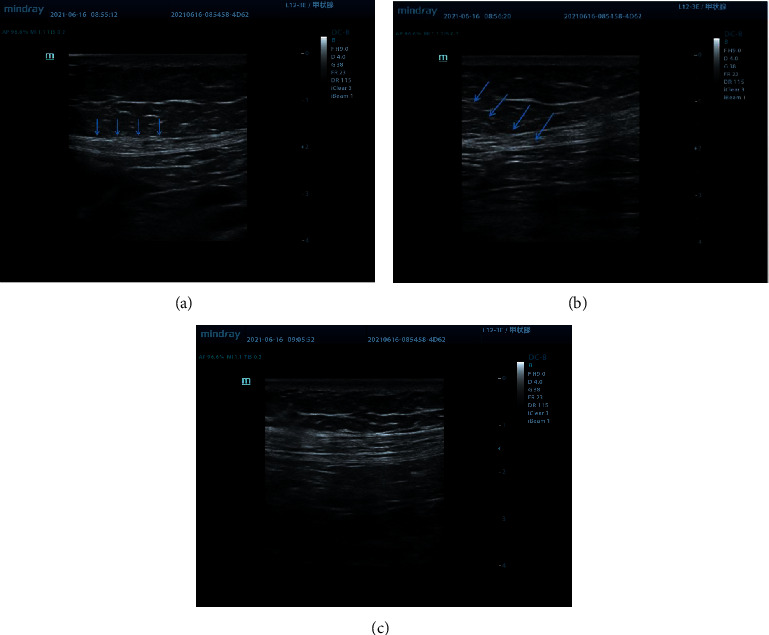
An example of PHN patient with MPS. (a) The MTP in the external oblique muscle of the abdomen. (b) The arrow shows the dry needle body. (c) The original MTP location after DN treatment.

All data were analyzed using SPSS 19.0 (IBM Corporation, Armonk, NY, USA), measurement data with normal distribution presented as the mean ± SD, and the independent sample *t*-test was used to compare the data between groups. Otherwise, median (quartile) was used for statistical description and the Mann–Whitney–Wilcoxon test was used for comparison between groups. The chi-squared test was used for statistical analysis of the categorical data which presented as percentage between groups. *P* value <0.05 was considered to represent a statistically significant difference, as shown in Figure 2.

## 3. Results

### 3.1. Comparison of Effective Rate between Group D and Group P

**Figure 3:**
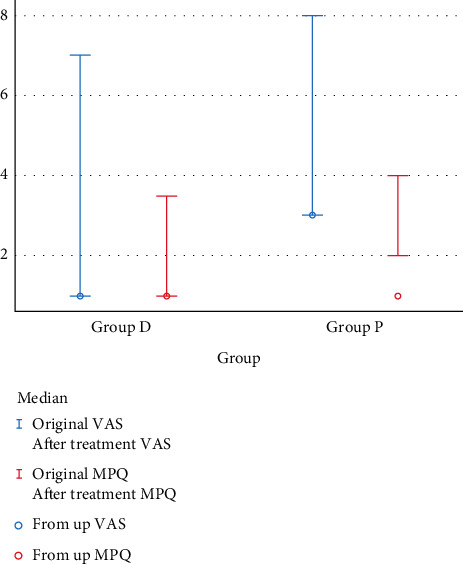
Comparison of the median of VAS and MPQ.

Compared with group P, the VAS and MPQ of group D decreased significantly (*p* < 0.05), as given in Table 2 and Figure 3.

### 3.2. Safety Evaluation

The main reference included dizziness, nausea, and vomiting. No serious adverse reactions occurred in both groups. No obvious nausea and vomiting were found in both groups. In group D, 6 patients (21.4%) needed to use odd other treatments, while for group P, the number was 14 (58.3%). The more details are given in Table 3.

## 4. Conclusion

Ultrasound-guided DN can be safely applied to the PHN patients mixed with MPS. DN and intradermal needling combined with press needling were more effective than only pharmacotherapeutic treatment for PHN mixed with MPS, with lower recurrent rate and higher patient's satisfactory rate.

## 5. Discussion

The safety and effectiveness of treatment have always been the concerns of pain specialist. PHN is the most common and intractable complication after herpes zoster infection, especially in the elderly. Its clinical features are peripheral neuralgia pain hypersensitivity, which leads to a serious impact on the quality of life [12]. However, there is no unified standard methods to eliminate this pain; unfortunately, this pain is easy to be recurrent [13]. In most pain departments, the routine treatment was oral pregabalin and mecobalamin; sometimes, paravertebral nerve block was performed in the spinal cord segment corresponding to the distribution area of herpes [14, 15]. Conventional treatment has an ideal therapeutic effect on PHN patients without MPS, but the therapeutic effect on PHN patients with MPS is not clear. Unfortunately, some studies have shown that more than a half of PHN patients had MPS [8]. This study showed that for these patients, DN treatment on PHN mixed with MPS had an unexpected good effectiveness, the effective rate was as high as 92.9%, and the recurrent rate was as low as 7.1% in the follow-up study three months later after the treatment. We found that ultrasound-guided DN is safe and effective in the treatment of PHN mixed with MPS.

MPS is a common clinical disease caused by MTrPs, but the mechanism of MTrPs formation is not very clear, and there are no universally accepted diagnostic methods. It is generally told that MTrPs may be caused by muscle trauma, long-term posture, or repeated local injury [16]. Simons [17] suggested that the abnormal endplate leads to trigger point formation which is caused by excessive acetylcholine (ACh). Gerwin [18] concluded that the excessive ACh could lead to local ischemia and hypoxia. Ischemia and hypoxia tissue lead to edema and inflammatory factors to release, which may sensitize dorsal horn neurons and supraspinal structures. MTrPs can be divided into active trigger points (ATrPs) and latent trigger points (LTrPs). LTrPs have all the characteristics of ATrPs except that it does not cause pain for the time being. It is more hidden and harmful. It does not cause spontaneous pain, but can cause neuromuscular and skeletal diseases, leading to changes in body posture. In the past 20 years, according to clinical practice and studies [17–22], clinical diagnosis has been constantly revised, but there is still no objective and reliable diagnostic criteria for LTrPs. It was the first time to use the overall targeted assessment method to infer the corresponding muscle fascia and further to verify the damaged muscle and muscle fascia by ultrasound in this study.

The potential role of DN is now reviewed from four different aspects in handling the activation of MTrPs: the spontaneous electrical activity (SEA), local ischemia and hypoxia, peripheral, and central sensitization. Both Hsieh et al. [23] and Sato et al. [24] demonstrated that DN may influence SEA when LTRs were elicited. There are several mechanisms to explain the response of local muscles to blood flow during DN stimulation, but the most credible is the release of vasoactive substances which leads to vasodilatation in small vessels and increased blood flow [25]. In addition, LTRs can significantly reduce the concentration of substance P (SP) and calcitonin gene-related peptide (CGRP) which can produce pain and peripheral sensitization. The pain produced by DN stimulates the afferent nerve fibers, which activates the supraspinal and higher central nervous systems involved in pain processing [26]. Different mechanisms can occur individually or simultaneously.

Ultrasound is one of the most widely used means in diagnosis and treatment. Studies have shown that MTrPs presented different ultrasonic echoes in different tissues [27, 28]. It suggested that the MTrPs were oval hypoechoic regions and also believed that MTrPs were hyperechoic regions. In one word, MTrPs were different under ultrasound images between the surrounding normal tissue. In this study, the following four steps were used to diagnose and treat MTrPs as shown in Figures 2(a)–2(c). The first step: the whole inspection, use the posture assessment to determine whether there was a significant posture change and then identify where MTrPs may be. The second step: carefully palpate the area where pain may occur and identify MTrPs. If the patients had obvious tenderness, pain, and local convulsions, then mark the corresponding areas. The third step: place the high-frequency probe in the center of the marked area and determine the heterogeneous area in the muscle or fascia by two-dimensional ultrasound scanning. The structure of muscle bundle membrane, extramuscular membrane, and intermuscular membrane is disordered and in cloud shape. On the contrast, the health fascicular membrane is orderly arranged. The fourth step: with real-time ultrasound-guided and the in-plane technology, use the disposable stainless steel needle (50 mm^*∗*^0.3 mm) to puncture the MTrPs area repeatedly; when MTrPs were inactivated, the entangled muscle fascia stretched immediately on the ultrasound image, and the echo of high brightness area decreased significantly. The morphological change of deformed muscle fascia caused by DN is the clinical basis for treatment of PHN mixed with MPS [29].

It is safe and effective to treat herpes zoster complicated with MPS by ultrasound-guided whole targeted DN combined with intradermal needling. Compared with pharmaceuticals only treatment, it can significantly improve the effective rate and reduce the recurrence rate. Ultrasound can supply the accuracy of diagnostic and treatment in PHN [30].

The limitations of this study are as follows: (1) this study only followed up the recurrent rate of three months after treatment. In view of the refractory and high recurrent characteristics of PHN, the next step of the study should be more long-term observation and follow-up. (2) The treatment effect has a cumulative effect, so it is impossible to distinguish the effect of each treatment. Diagnosis and treatment are still in the exploratory stage and more experience needs to be accumulated in the next step.

## Figures and Tables

**Table 1:** Comparison of the baseline level and the clinical characteristics.

Items	D group (*n* = 28)	P group (*n* = 26)	*t*/*χ*^2^/*Z*	*P* value
Age (years)	69.5 (59.3–76.5)	70.0 (64.0–78.5)	0.26	0.98
Weight (kg)	61.8 ± 9.1	58.4 ± 12.3	0.29	0.77
Height	161.5 ± 6.7	162.0 ± 6.8	1.17	0.25
BMI	23.5 ± 2.4	22.1 ± 3.7	0.68	0.10
Gender (female)	16 (57.1%)	16 (61.5%)	0.11	0.79
Duration of PHN (days)	15.0 (10.5–23.3)	17.0 (13.5–19.0)	1.09	0.27
VAS	7.0 (6.3–8.0)	8.0 (7.0–8.0)	0.98	0.33
MPQ	3.5 (3.0–4.0)	3.0 (3.0–4.0)	0.46	0.65
Internal diseases				
Cardio diseases (%)	15 (53.6%)	17 (65.3%)	0.78	0.42
Respiratory diseases (%)	9 (32.1%)	7 (26.9%)	0.17	0.77
Diabetes diseases (%)	7 (21.4%)	6 (19.2%)	0.03	1.00

**Table 2:** Comparison of the therapeutic effectiveness.

Items	Group D (*n* = 28)	Group P (*n* = 26)	*t/χ* ^*2*^ */Z*	*P* value
The first-week effective rate	10 (35.7%)	0 (0%)	11.18	<0.01
The fourth-week effective rate	19 (67.9%)	0 (0%)	58.74	<0.01
The one-month effective rate	26 (92.9%)	10 (38.5%)	17.95	<0.01
The two-month effective rate	20 (71.4%)	7 (26.9%)	58.74	<0.01
The three-month recurrence rate	2 (7.1%)	9 (34.6%)	6.18	0.02

The VAS score <3 and/or the MPQ score <2 represents effective treatment. The VAS score >3 or the MPQ score >2 represents recurrence.

**Table 3:** Odd other treatment's rate and satisfactory rate.

Group	Group *D* (*n* = 28)	Group *P* (*n* = 26)	*t/χ* ^*2*^ */Z*	*P* value
Odd other Treatments (*n*, %)	6 (21.4%)	14 (53.8%)	6.01	0.02
Satisfactory Rate	24 (85.7%)	4 (15.4%)	26.71	<0.01

## Data Availability

The data used to support the finding of this study are available from the corresponding author upon request.

## Supplementary Materials

Supplementary Materials The supplementary material for this article are included within the supplemental file.
